# Supplementary material for: Novel Triterpenic Acid—Benzotriazole Esters Act as Pro-Apoptotic Antimelanoma Agents
Source: Int J Mol Sci. 2022 Sep 1;23(17):9992. doi: 10.3390/ijms23179992 (PMC9456456; doi:10.3390/ijms23179992)
Supplement: Supplementary file 1 [file ijms-23-09992-s001.zip › ijms-1892157-SI.pdf]

SUPPLEMENTARY MATERIAL

Novel Triterpenic Acid – Benzotriazole Esters act as Pro-apoptotic Antimelanoma Agents

Marius Mioc [1,2], Alexandra Mioc [2,4,*], Alexandra Prodea [1,2], Andreea Milan [1,2], Mihaela Balan-Porcarasu [3], Roxana Racoviceanu [1,2], Roxana Ghiulai [1,2], Gheorghe Iovanescu [5], Ioana Macasoi [2,6], George Draghici [2,6], Cristina Dehelean [2,6] and Codruta Soica [1,2]

[1] Department of Pharmaceutical Chemistry, Faculty of Pharmacy, "Victor Babes" University of Medicine and Pharmacy Timisoara, Eftimie Murgu Sq., No. 2, 300041 Timisoara, Romania
[2] Research Centre for Pharmaco-Toxicological Evaluation, "Victor Babes" University of Medicine and Pharmacy Timisoara, Eftimie Murgu Sq., No. 2, 300041 Timisoara, Romania
[3] Institute of Macromolecular Chemistry 'Petru Poni', Iasi, Romania
[4] Department of Anatomy, Physiology, Pathophysiology, Faculty of Pharmacy, "Victor Babes" University of Medicine and Pharmacy Timisoara, Eftimie Murgu Sq., No. 2, 300041 Timisoara, Romania
[5] Department of Otolaryngology, Faculty of Medicine, "Victor Babes" University of Medicine and Pharmacy Eftimie Murgu Sq., No. 2, 300041 Timisoara, Romania
[6] Department of Toxicology, Faculty of Pharmacy, "Victor Babes" University of Medicine and Pharmacy Timisoara, Eftimie Murgu Sq., No. 2, 300041 Timisoara, Romania
* Correspondence: alexandra.petrus@umft.ro

Abstract: Pentacyclic triterpenes like betulinic, ursolic and oleanolic acids are currently reported as efficient and selective anticancer agents whose underlying mechanisms of action have been widely investigated. The introduction of N-bearing heterocycles such as triazoles into the structure of natural compounds, especially pentacyclic triterpenes, has proved to yield semisynthetic derivatives with increased antiproliferative potential as opposed to the unmodified starting compounds. In this work, we report the synthesis and biological assessment of benzotriazole esters of BA, OA and UA (compounds **1-3**). The esters were obtained in moderate yields (28-42%). All three compounds showed a dose-dependent reduction in cell viability against A375 melanoma cells and no cytotoxic effects against healthy human keratinocytes. Morphology analysis of treated cells showed characteristic apoptotic changes consisting of nuclear shrinkage, condensation, fragmentation, and cellular membrane disruption. Western blot and rtPCR analysis reinforced the proapoptotic evidence showing a reduction in anti-apoptotic Bcl-2 expression and upregulation of the pro-apoptotic Bax and caspase 9. High resolution respirometry studies showed that all three compounds were able to significantly inhibit mitochondrial function. Molecular docking showed that compounds **1-3** showed an increase in binding affinity against Bcl-2 as opposed to BA, OA and UA and similar binding patterns compared to known Bcl-2 inhibitors.

<u>**Contents**</u>

Figure S1. 1H NMR spectra of compound **1**
Figure S2. 13C NMR spectra of compound **1**
Figure S3. 1H NMR spectra of compound **2**
Figure S4. 13C NMR spectra of compound **2**
Figure S5. 1H NMR spectra of compound **3**
Figure S6. 13C NMR spectra of compound **3**
Figure S7. FTIR spectra of compound **1**
Figure S8. FTIR spectra of compound **2**
Figure S9. FTIR spectra of compound **3**

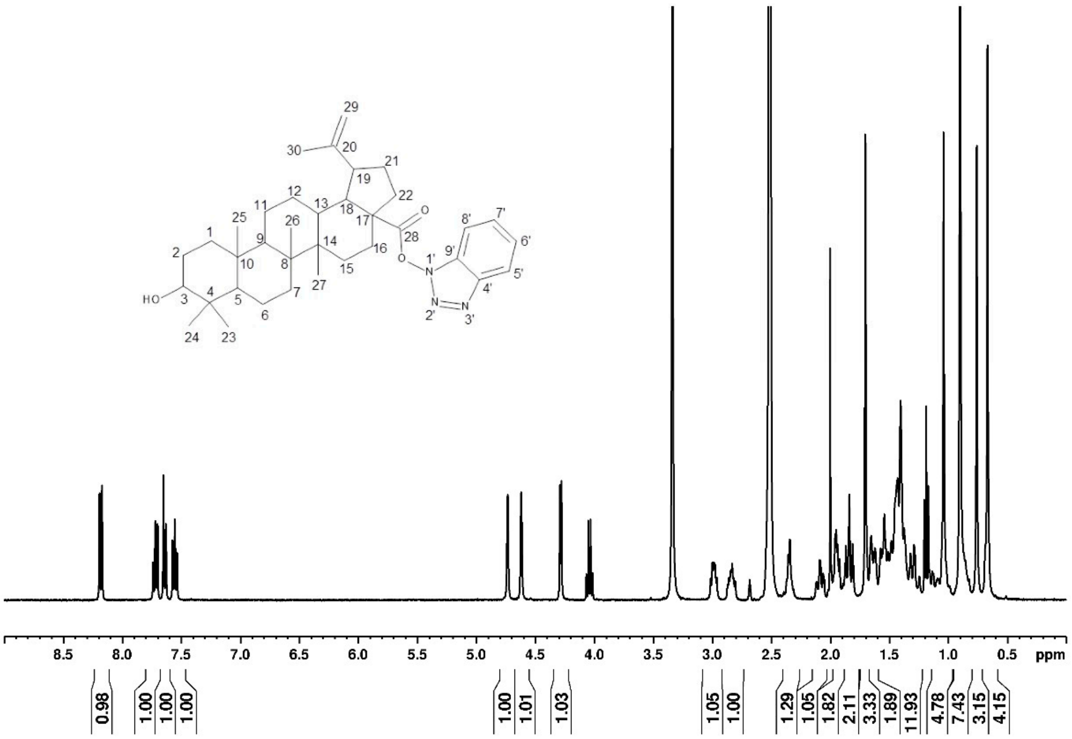

Figure S1. ^{1}H NMR spectra of compound **1**

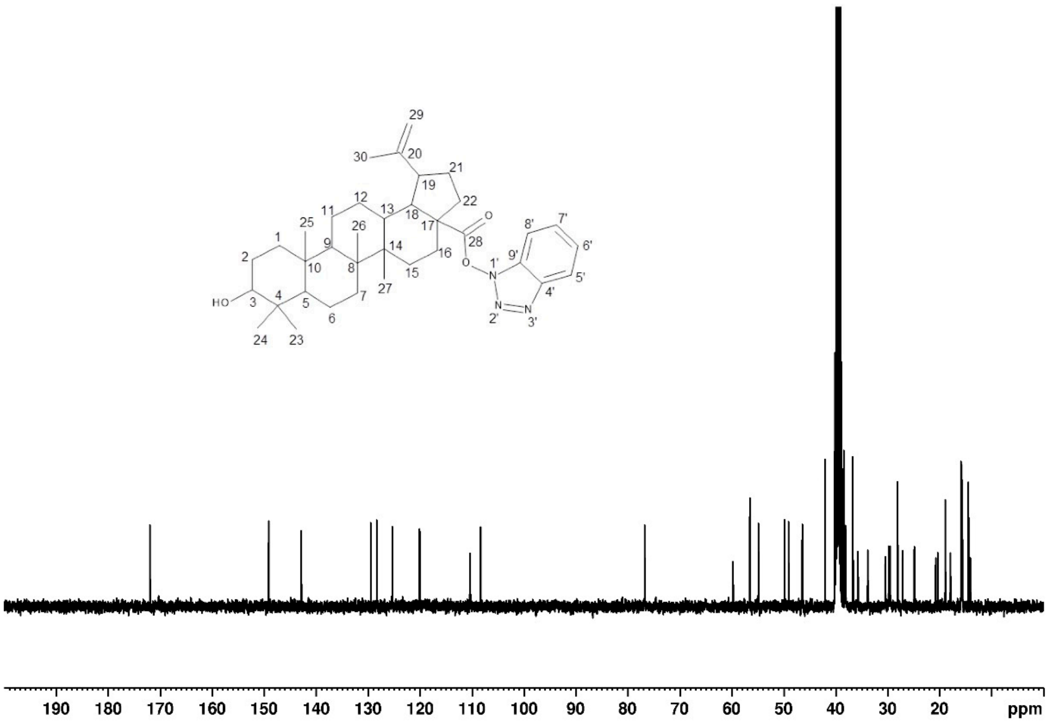

Figure S2. ^{13}C NMR spectra of compound **1**

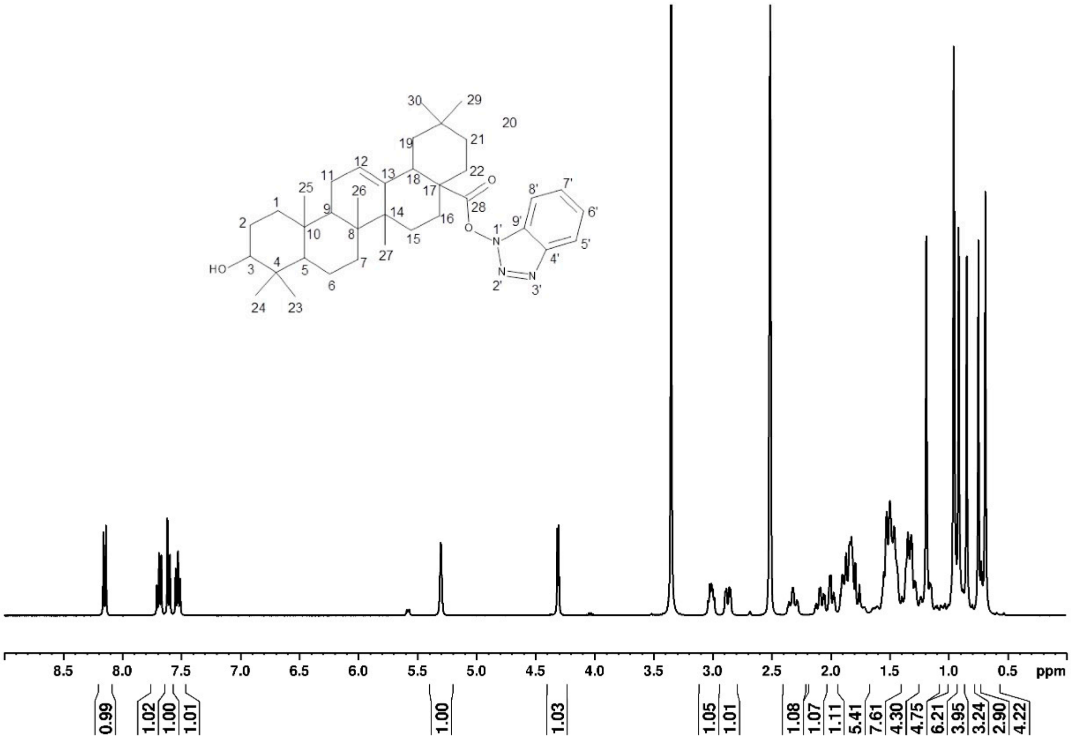

Figure S3. ^{1}H NMR spectra of compound **2**

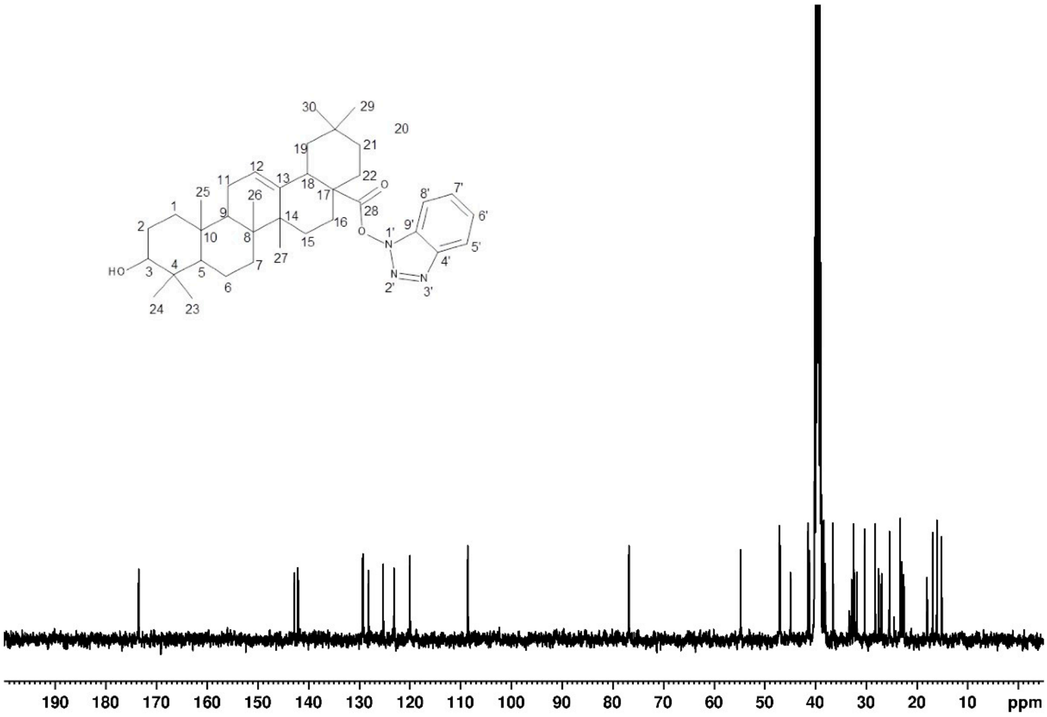

Figure S4. ^{13}C NMR spectra of compound **2**

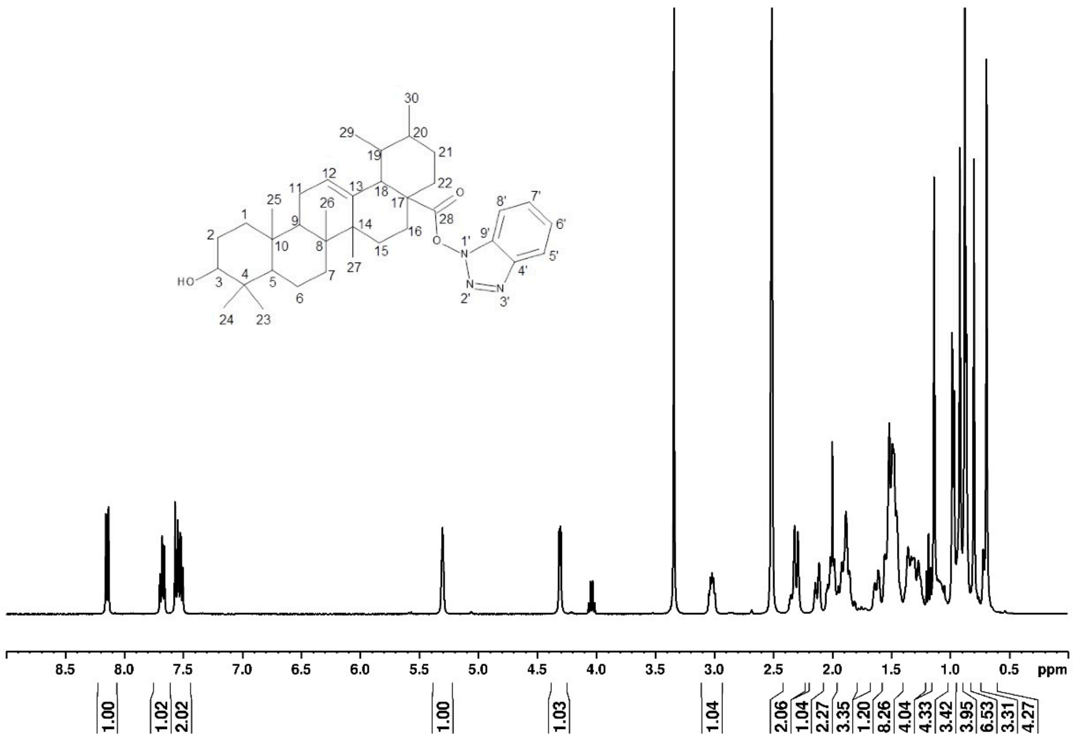

Figure S5. ¹H NMR spectra of compound **3**

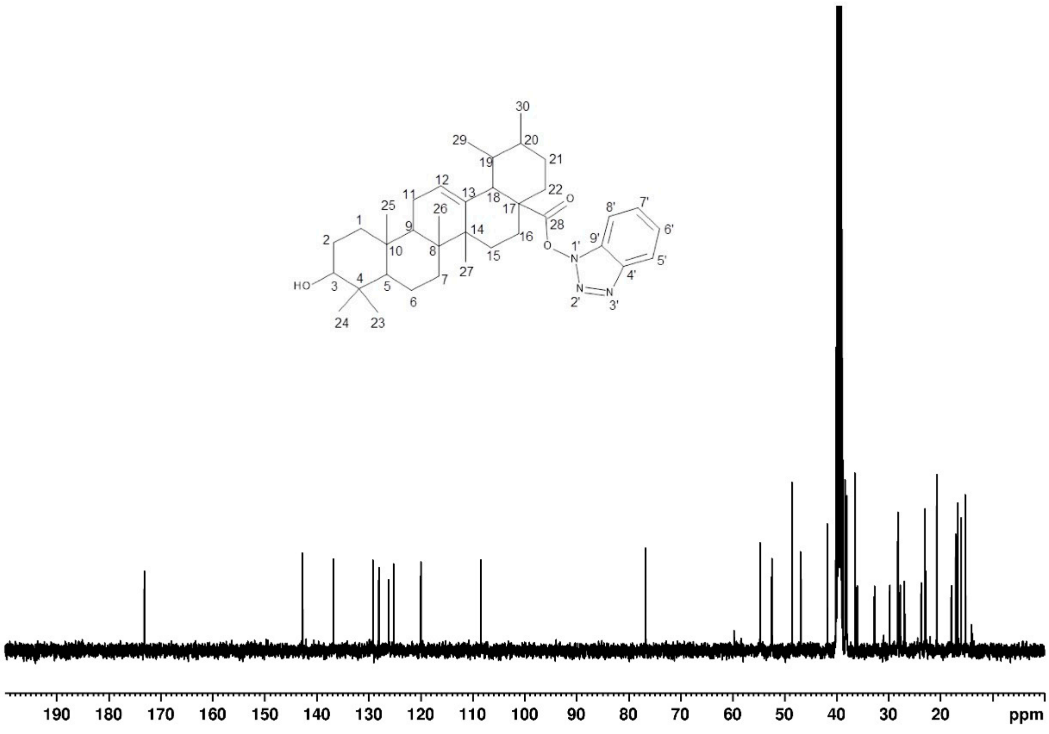

Figure S6. 13C NMR spectra of compound **3**

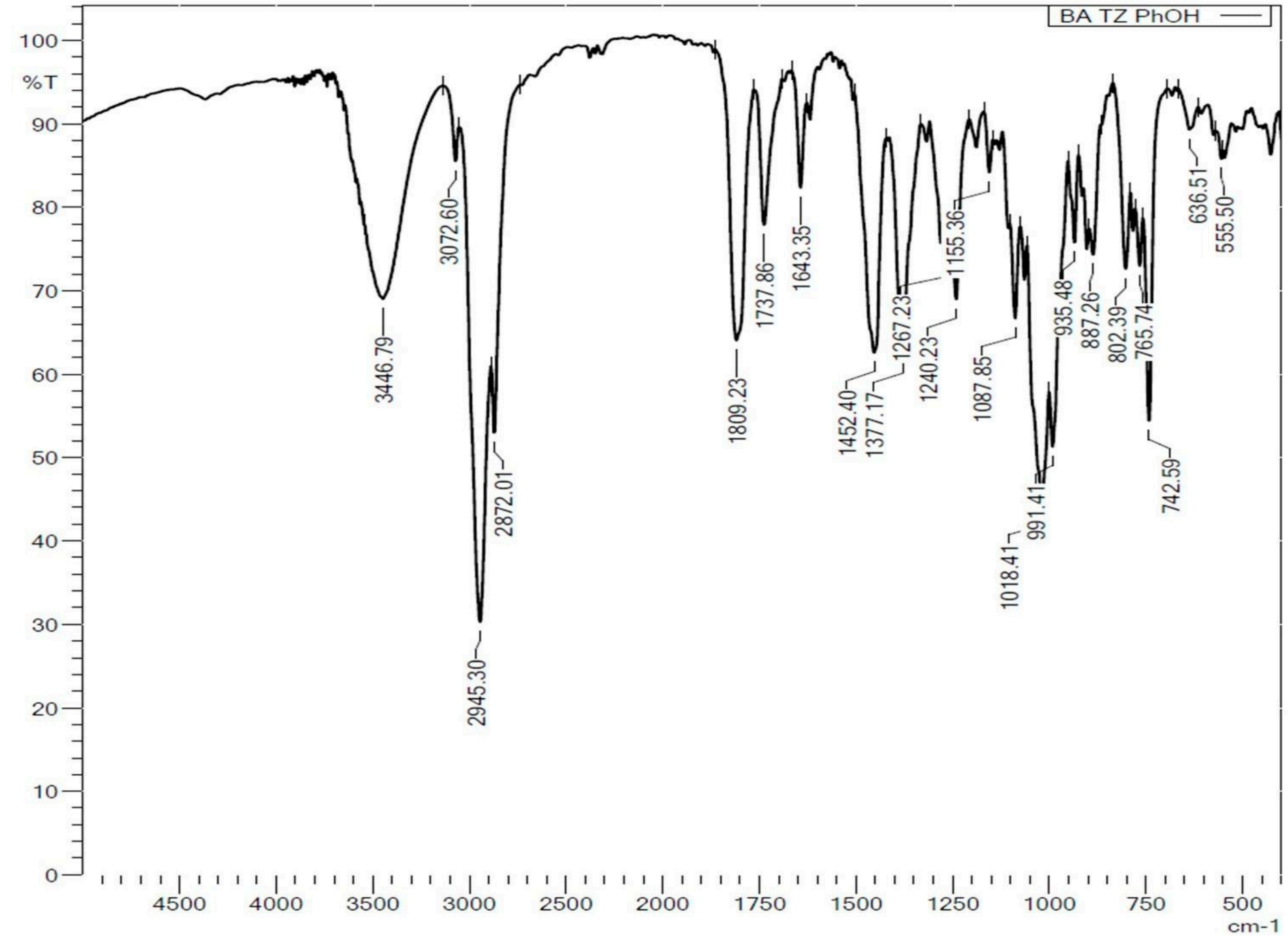

Figure S7. FTIR spectra of compound **1**

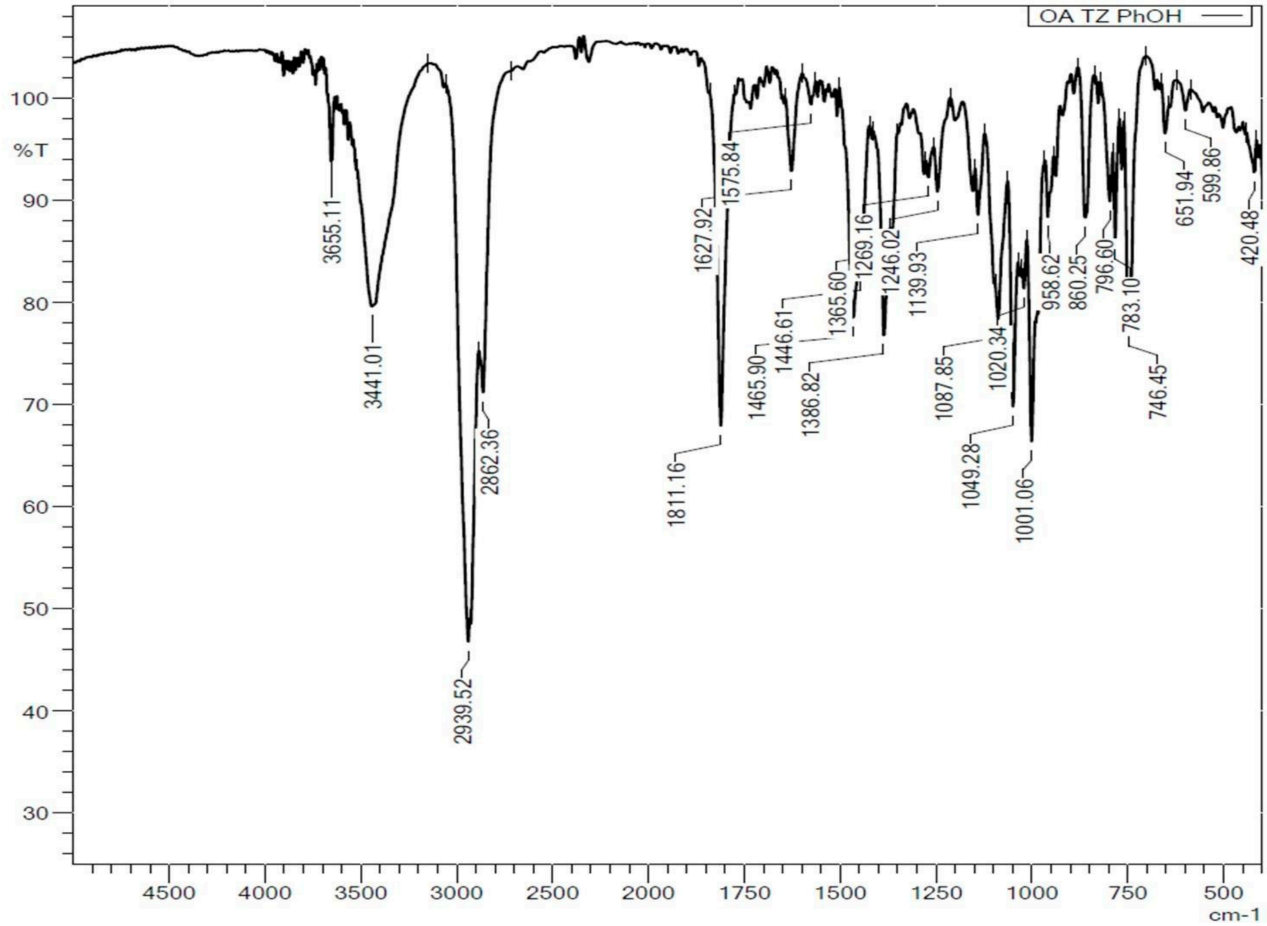

Figure S8. FTIR spectra of compound **2**

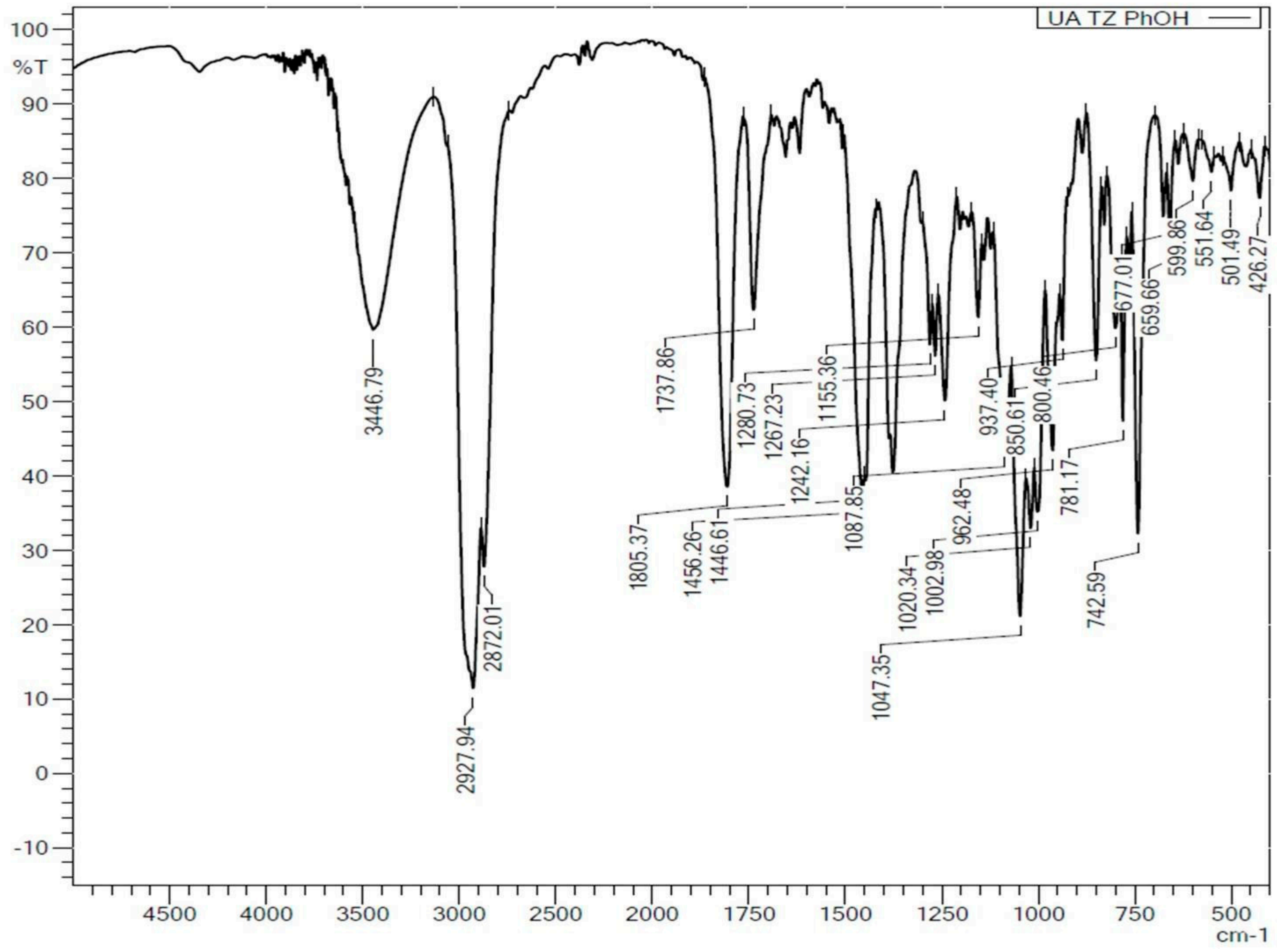

Figure S9. FTIR spectra of compound **3**
